# Canadian Consensus Recommendations for the Management of Operable Stage II/III Non-Small-Cell Lung Cancer: Results of a Modified Delphi Process

**DOI:** 10.3390/curroncol30120755

**Published:** 2023-12-06

**Authors:** James Tankel, Jonathan Spicer, Quincy Chu, Pierre Olivier Fiset, Biniam Kidane, Natasha B. Leighl, Philippe Joubert, Donna Maziak, David Palma, Anna McGuire, Barbara Melosky, Stephanie Snow, Houda Bahig, Normand Blais

**Affiliations:** 1 Department of Thoracic Surgery, McGill University Health Center, Montreal, QC H3G 1A4, Canada; 2 Department of Medical Oncology, Cross Cancer Institute, Edmonton, AB T6G 1Z2, Canada; 3 Department of Pathology, McGill University Health Center, Montreal, QC H3G 1A4, Canada; 4 Section of Thoracic Surgery, University of Manitoba & Cancer Care Manitoba, Winnipeg, MB R3A 1R9, Canada; 5 Division of Medical Oncology, Princess Margaret Cancer Center, Toronto, ON M5G 2C4, Canada; 6 Department of Pathology, Institut Universitaire de Cardiologie et de Pneumologie de Québec-Université, Laval, QC G1V 4G5, Canada; 7 Department of Thoracic Surgery, Ottawa Hospital, Ottawa, ON K1Y 4E9, Canada; 8 Department of Radiation Oncology, London Health Services Center, London, ON N6A 5A5, Canada; 9 Department of Thoracic Surgery, Vancouver General Hospital, Vancouver, BC V5Z 1M9, Canada; 10 Department of Medical Oncology, BCCA, Vancouver, BC V5Z 4E6, Canada; 11 Department of Medical Oncology, Queen Elizabeth II Health Sciences Center, Halifax, NS B3H 3A7, Canada; 12 Department of Radiation Oncology, Centre Hospitalier de l’Université de Montréal, Montreal, QC H2X 3E4, Canada; 13 Department of Medical Oncology, Centre Hospitalier de l’Université de Montréal, Montreal, QC H2X 3E4, Canada

**Keywords:** non-small-cell, lung cancer, neoadjuvant, immunotherapy, consensus guidelines, Delphi

## Abstract

The treatment paradigm for patients with stage II/III non-small-cell lung cancer (NSCLC) is rapidly evolving. We performed a modified Delphi process culminating at the Early-stage Lung cancer International eXpert Retreat (ELIXR23) meeting held in Montreal, Canada, in June 2023. Participants included medical and radiation oncologists, thoracic surgeons and pathologists from across Quebec. Statements relating to diagnosis and treatment paradigms in the preoperative, operative and postoperative time periods were generated and modified until all held a high level of consensus. These statements are aimed to help guide clinicians involved in the treatment of patients with stage II/III NSCLC.

## 1. Introduction

Lung cancer is the leading cause of cancer-associated mortality worldwide, accounting for approximately 20% of all cancer-related deaths [1]. In Canada alone, there were 233,900 new diagnoses in 2022 with an associated 85,100 deaths [2]. Many of these cases are non-small-cell lung cancer (NSCLC), as this subtype is found in approximately 85% of patients [3]. The lack of typical symptoms in earlier stages of disease often leads to lung cancer being diagnosed at an advanced stage. As such, whilst screening programs have increased the number of early lung cancer diagnoses [4], 50% of those diagnosed with lung cancer have metastatic disease at presentation [5]. Population-level studies from Canada have shown that stage I, II and III lung cancer represent 20%, 8% and 19%, respectively, of all NSCLC diagnoses [6].

Significant breakthroughs in the treatment of NSCLC have resulted in the diagnostic and therapeutic landscape becoming increasingly complex, particularly for patients with stage II/III disease. These breakthroughs are based upon improvements in staging due to easier access to PET scanning and endobronchial ultrasound in addition to a better understanding of how biomarkers and genetic mutations influence treatment decisions. Taken together, these drive a rapidly evolving paradigm of precision systemic therapies. As a result, novel neoadjuvant, perioperative and adjuvant strategies have been developed incorporating chemotherapy, immune-checkpoint inhibitors and targeted agents in various combinations and sequences. The key goal is to prolong survival by reducing the rates of disease recurrence which affects 50% of patients with stage II disease and 60% of patients with stage III NSCLC [7].

The current standard of care in Canada for patients with stage II/III is broadly aligned with the American Society of Clinical Oncology [8], the National Comprehensive Cancer Network [9] and the European Society of Medical Oncology [10] guidelines and typically involves multimodal therapy. However, stage II/III NSCLC incorporates a heterogeneous spectrum of disease with equally diverse treatment options. In addition, the survival benefit associated with both local and systemic treatment varies between different treatment modalities and stages of disease [11].

To provide some clarity to the evolving management of patients with stage II/III NSCLC, we undertook a pan-Canadian modified Delphi process integrating the opinions of experts from medical and radiation oncology, pathology and thoracic surgery. Based on this process, we have generated comprehensive consensus recommendations for the management of patients with operable stage II/III NSCLC that incorporate diagnostic and neoadjuvant treatment and operative, pathologic and adjuvant treatment elements applicable in the Canadian public healthcare system.

## 2. Materials and Methods

### 2.1. Consensus Panel

In May 2023, a working group of three experts was assembled which included an attending thoracic surgeon, medical oncologist and radiation oncologist (JS, NB, HB) and a thoracic surgery fellow (JT). During the course of several virtual meetings, a preliminary draft of the recommendations was generated in keeping with the PICO format (population, patients or problem; intervention; comparison; and outcome). The statements incorporated four major themes: (1) diagnosis and pre-operative assessment; (2) neoadjuvant systemic immuno-chemotherapy; (3) operative considerations; and (4) post-operative management.

**Figure 1:**
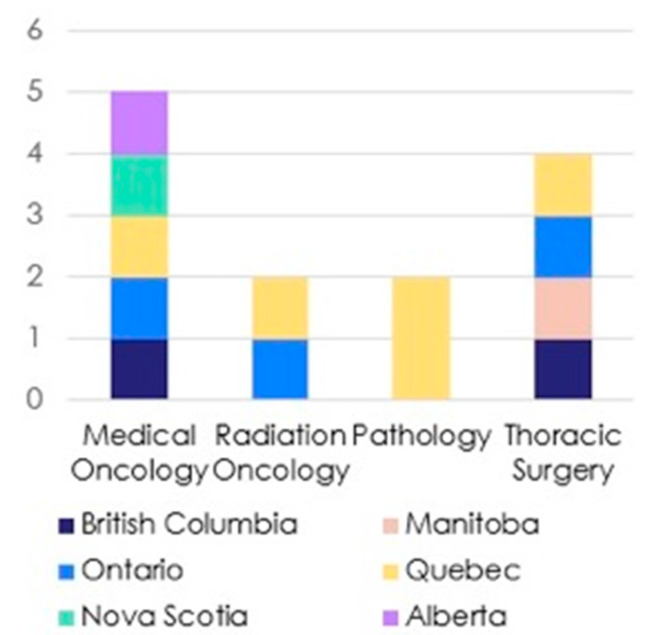
A graphical representation of speciality and region of Delphi panel members.

Once the recommendations had been developed, an online modified Delphi process was conducted during which the statements were sent independently to a committee of eleven published and recognized experts from across Canada within the fields of thoracic surgery, medical and radiation oncology and pathology, as shown in Figure 1. Due to different training paradigms and practice patterns in Canada, several of the thoracic surgeons (BK, AM) also had expertise in interventional pulmonology domains (high volume EBUS, primary involvement in diagnosis and staging patients with lung cancer). The Delphi panel members and expert panel (except JT) were invited via secure institutional e-mail to anonymously rate the recommendations using a Likert score (1—extremely agree, 2—agree, 3—neither agree nor disagree, 4—disagree and 5—extremely disagree) and submit free-text comments to an expert reviewer (JT). Recommendations were updated iteratively based on Delphi panel feedback before the next round of voting. The remaining members of the expert working group were blinded to the process of statement modification.

In the second and third Delphi rounds, only recommendations with a consensus <75% or with a decrease in the level of agreement in comparison to the previous Delphi round were included. For the recommendations presented, the cumulative median score, range and percentage of anonymized panelists agreeing with the statement during the previous round were presented to the panel members alongside each amended statement as well as the previous iteration. Panelists could recuse themselves from responding to particular statements if they reported that the topic covered was beyond the scope of their specialty. Panelists were given one week to complete each Delphi round. A reminder was sent via e-mail on the penultimate day of the week and a response level of >80% was required before proceeding to the next round.

In total, three modified Delphi rounds were held, followed by an in-person meeting of the expert working group and Delphi panelist members in Montreal, Canada, in June 2023. Each statement was reviewed and attendees were invited to discuss points they felt pertinent. Statements identified as having further scope for refinement with a view to achieving a greater level consensus were modified with panelists asked to score their level of agreement for the updated statement in a post-meeting online follow-up via the same e-mail-based online portal. These modifications were used to refine specific points but not to change the spirit of the statement.

The consensus statements were subsequently presented at the Early-stage Lung cancer Internation eXpert Retreat (ELIXR23) meeting held in Montreal, Canada, in June 2023, which included a broader array of specialists involved in all aspects of the management of NSCLC who were also invited to comment and identify shortfalls in the proposed consensus statements. Whilst these comments were not incorporated into the statements themselves, they were used to formulate elements of the discussion below.

### 2.2. Literature Review and Data Synthesis

For each PICO question, the lead authors (JT, JS and NB) searched EMBASE and Medline for relevant literature. All English language full articles published between 1 January 1990 and 1 October 2023 were included. The bibliographies of relevant texts were also searched in order to reveal any additional texts that may be pertinent. Additionally, recent and relevant publications included conference proceedings and abstracts identified by the expert working group and the wider Delphi panel.

Members graded the class of recommendation according to the American Association of Thoracic Surgeons position statement on creating clinical guidelines [12]. The American Heart Association clinical practice guidelines classification system was used to grade the quality of supporting evidence [13]. Briefly, this is classified as: Level A, high quality data from multiple randomized trials; level B-R (randomized), data from a single randomized trial; level B-NR (non-randomized), high quality, non-randomized data; level C-LD (limited data), observational or registry-level data; and level C-EO (expert opinion), based on consensus or expert opinion.

## 3. Results

**Figure 2:**
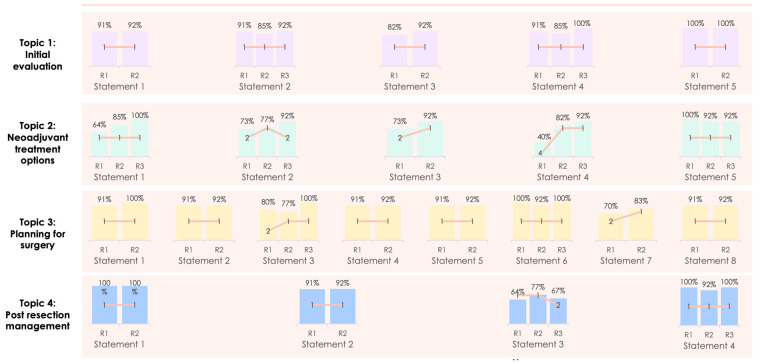
Changing consensus of each statement through each Delphi stage.

**Figure 3:**
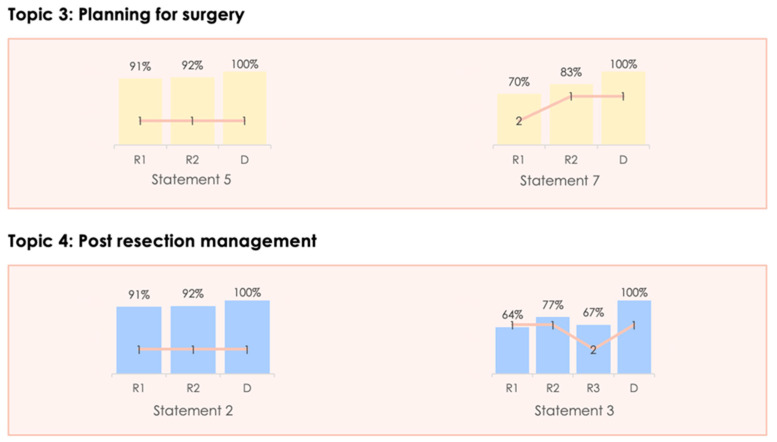
Changing consensus for the statements changed following the in-person meeting.

During the Delphi process, three electronic rounds were held before the face-to-face meeting. The change in the level of consensus between each Delphi stage is shown in Figure 2, whilst the four statements felt to have scope for refinement that underwent a further Delphi stage following the in-person meeting are described in Figure 3. As can be seen, a high level of consensus was ultimately achieved for all of the proposed statements. Participation in each round was 11/13 (84.6%), 13/13 (100%), 12/13 (92.3%) and 12/13 (92.3%) for the first, second, third and fourth rounds respectively. The summary of the recommendations are described below(Table 1, Table 2, Table 3 and Table 4). 

## 4. Discussion

### 4.1. Staging and Diagnosis

As shown in the statements made in Table 1, accurate clinical staging of NSCLC is central to formulating treatment decisions. Computed tomography (CT), which is often the initial examination performed, should ideally include the upper abdomen and be performed with intravenous contrast. This aids with identifying extrapulmonary invasion, better delineates hilar and mediastinal lymphadenopathy as well as pulmonary vascular anatomy and in revealing metastatic disease in the upper abdomen and bones [14].

Many new diagnoses of lung cancer originate from lung cancer screening programs. Typically, these programs use low-dose, non-contrast-infused CT scanning. There is no specific evidence concerning the added benefit of repeating a CT scan with contrast infusion, and as such, the requirement to perform another scan is likely to depend on the location, size and the anatomy adjacent to the lesion, particularly if there is concern regarding the resectability. A concern of bulky mediastinal lymphadenopathy should also prompt repeat imaging to be performed.

In addition to CT, ^18^F-FDG PET/CT is an invaluable tool in the staging of patients with NSCLC. This modality has several advantages, including that increased metabolic activity of the primary lesion is negatively associated with survival and suggests more aggressive biology of the tumor with a greater risk of lymph node metastases [15]. There is also some evidence that suggests a correlation between the primary tumor avidity and response to induction immuno-chemotherapy [16]. The use of PET/CT also improves the determination of the extent of local disease. In one study, PET/CT correctly predicted the T stage in 86% of cases of NSCLC compared to 68% with CT [17]. Adding ^18^F-FDG PET/CT to a contrast-infused CT scan seems to provide more accurate staging than either modality alone as PET/CT also outperforms CT in terms of identifying distant metastatic disease [18,19,20,21]. Therefore, if PET/CT fails to show evidence of sub-diaphragmatic disease, repeating a contrast-infused CT abdomen is not necessary.

As demonstrated in randomized data, the addition of PET-CT also improves the identification of mediastinal lymphadenopathy [22]. For example, using a cut-off of >10 mm, the pooled sensitivity and specificity of contrast-enhanced CT in identifying mediastinal lymphadenopathy is 55% and 81%, respectively [23]. Conversely, PET-CT has been found to have a sensitivity of 90.5% and specificity of 60.5% with an associated negative predictive value of 83.3% for metastatic mediastinal lymphadenopathy [24]. Whilst the incidence of false positivity varies greatly from 12 to 40% [23], a range somewhat explained in areas of the world with a high prevalence of granulomatous or inflammatory mediastinal disease [25], a more conservative false-negative rate of 14–16% is commonly described [26,27]. Despite the benefits of this imaging modality, resource availability may limit access to PET-CT in some regions implying that a case-by-case approach is probably more appropriate with PET-CT being preferentially used for those with suspicion of metastatic disease or mediastinal lymphadenopathy.

These results highlight the importance of obtaining a tissue diagnosis from mediastinal lymph nodes with endobronchial and/or esophageal ultrasound (EBUS/EUS) with transbronchial needle aspiration and, when required, mediastinoscopy in stage II/III NSCLC. As these modalities are equivalent in terms of detecting mediastinal metastases with a sensitivity of 79–81% and diagnostic accuracy of 93% [28], there is a preference towards performing EBUS which is less morbid, performed as an outpatient procedure and more cost effective [29]. In addition, a positive EBUS of mediastinal lymph nodes can replace CT guided transthoracic biopsy of the primary lesion.

However, approximately 14% of patients will have false-negative staging of N2 mediastinal nodes irrespective of whether EBUS or mediastinoscopy is chosen [30]. Whilst it is suggested that all suspicious nodes on cross-sectional imaging be sampled sequentially at N3, N2 and N1 stations [31], approximately 30% of endoscopic procedures have been described as ‘insufficient’, demonstrating the operator-dependent nature of EBUS/EUS [32]. As such, mediastinoscopy may still be required if this modality is not available or in the setting of a negative or indeterminate result when there is high clinical suspicion of nodal metastasis. Routinely performing EBUS and mediastinoscopy together is associated with an improvement in negative predictive value by approximately 10%; however, data suggest that almost 30 patients need confirmatory mediastinoscopy to upstage one patient incorrectly classified as N0 [33].

Early detection of brain metastases alters treatment trajectory and improves survival [34]. Up to 20% of patients with suspected stage III NSCLC have brain metastases at baseline and contrast-infused MRI seems to be more sensitive at diagnosing smaller lesions than CT [35,36]. In a Dutch study, whilst 7% of patients with suspected stage III NSCLC had brain metastases seen on CT, a further 4.5% had lesions detected only with MRI [37]. However, major guidelines are conflicting with the American Joint Committee on Cancer guidelines recommending MRI as part of staging investigations for stage III disease only whilst the NCCN guidelines recommend MRI of the brain for those with clinical stage II disease as well [9,38]. One recent study found the incidence of brain metastases on MRI to be 8/171 (4.7%) for clinical stage II disease and 13.2% for clinical stage III disease [39]. With regards to histological subtype, compared to squamous cell carcinoma, adenocarcinoma has been found to be more commonly associated with brain metastases when compared stage by stage in neurologically asymptomatic patients [40]. However, the diagnostic yield of MRI is similar between histological subtypes and therefore we cannot suggest different imaging modalities based on the type of NSCLC alone [39]. Nevertheless, considering the limited access to MRI in some regions of Canada, we suggest prioritizing those with stage III disease and using CT where an MRI is not accessible.

In order to ensure these investigations are used appropriately, early communication between care providers is essential. Although a recent European study demonstrated the low cost associated with the diagnostic studies and procedures for patients with NSCLC [41], these costs vary between healthcare systems. Beyond financial considerations, access and availability to resources is also likely to vary between region and countries. As such, the decisions to utilize investigative strategies must be commensurate with the treatment expectations and trajectory of care for that patient.

In some institutions, this takes the form of a navigation meeting which focuses specifically on ensuring patients are progressing along the appropriate investigation pathways and being referred to treating physicians in a timely manner. Whilst not universally available, a specialist nurse coordinator has been found to improve metrics of information exchange, empathy and quality of life in terms of physical, social, emotional and familial well-being [42]. Data suggest that use of a specialist nurse to facilitate staging may also reduce the time needed to complete investigations [43].

Although not part of the staging pathway, biomarker testing with next-generation sequencing (NGS) is a key aspect of diagnosis and should be performed as early as possible to allow personalization of treatment decisions. Guidelines from the American College of Pathologists suggest this panel should include at a minimum EGFR, ALK, MET, BRAF, KRAS and ROS1 genes, although since this guideline has been written, approved targeted agents specific to RET, HER2, NTRK genes highlight the need to perform a broad NGS panel [44]. Other international organizations have supported a similar approach with an emphasis on testing for aberrations for which there is a specific implication in the treatment pathway [45,46]. The benefit of immunotherapy appears limited in most oncogene-addicted lung cancer subtypes, including ALK and ROS1 and potentially RET- and HER2-altered lung cancer [47].

Particularly with regards to ALK and ROS1, which are found at rates of 1.5% and 0.3%, respectively, in data from Quebec and in 1–7% of patients with NSCLC in other studies [48,49], immunotherapy is not thought to be effective. In addition, routine PD-L1 tumor expression should be performed as the level of expression is related to drug efficacy in the context of neoadjuvant systemic immunotherapy and may impact individual treatment decisions.

It is imperative that once these investigations are completed, the patient’s case be discussed in a multidisciplinary meeting. This should ideally include radiation and medical oncologists, pathologists, radiologists, nuclear medicine specialists, respirologists and thoracic surgeons. Diagnostic and therapeutic decisions originating from a multidisciplinary team have been shown to affect treatment pathways and improve survival outcomes and lead to better quality of life and closer adherence to national guidelines [50,51,52,53,54]. Nevertheless, inclusion of the patient as a stakeholder in the decision making with regards to their treatment is fundamental, particularly as patients may weigh disease survival and treatment-related symptoms differently from their clinicians [55]. Therefore, we recommend patients with stage II/III NSCLC discuss their treatment options with a thoracic surgeon, medical oncologist and radiation oncologist before deciding on how they would like to be treated so that the patient’s wishes can be included in the formation of the MDT recommendation. There are no clear guidelines regarding which patients need discussing within the MDT meeting and it is an aspirational goal to discuss all stage II/III patients. If time constraints do not permit this, in the absence of guidelines on which cases need discussion, priority should be given to those for whom there are treatment alternatives or uncertainty about how to best treat the patient.

Central to these discussions is the decision regarding resectability of the lesion and associated lymph nodes. A discussion regarding the definition of resectability is beyond the remit of these guidelines. However, central to the complex decision to perform surgery is a balance of patient preferences and operability vis-à-vis the ability of the surgeon to achieve an R0 resection and complete lymph node evaluation of one hilar and three mediastinal stations, a standard endorsed by the American College of Surgeons’ commission on cancer for curative-intent pulmonary resection [56]. To establish whether a patient’s disease is resectable, an initial consultation with a trained and experienced thoracic surgeon is required. Once resectability has been established, further discussion with the medical and radiation teams can take place to discuss neoadjuvant options and treatment alternatives.

### 4.2. Neoadjuvant Treatment Options

The consensus statements and the level of recommendation relevant to this section are described in Table 2. 

Data suggest a delay in starting definitive treatment is associated with an increase in cancer-associated mortality [57,58]. Such a delay could originate from lack of formal definition of ‘borderline resectability’. This is a surgeon- and center-specific term that encapsulates patients in whom a ‘complete resection’ may not be achievable [59]. However, beyond the operative metric of whether an R0 resection can be performed, the presence of N2 lymphadenopathy may also steer some patients towards non-operative treatments for several reasons. Firstly, the superiority of surgical resection in comparison to definitive chemoradiation has not yet been proven in those with N2 disease beyond highly selected patients identified in subgroups and nonrandomized studies [60,61,62,63,64]. Secondly, 65% of patients with cN2 disease have been found to have involvement of the nodal capsule, which has been associated with an increase in disease recurrence and poorer survival outcomes [65]. Thirdly, the anatomical location or size of a node may mean that a complete resection is not feasible [66].

However, it is important to note that data comparing neoadjuvant immuno-chemotherapy to chemoradiotherapy specifically among patients with N2 disease is not yet available and represents an important area of future research.

Practically, this means that the allocation of certain patients to different treatment algorithms varies from center to center and from patient to patient, depending on the surgeon’s ability to perform a complete resection and the willingness of the patient to undergo surgery. It is imperative that these center-, surgeon- and patient-specific nuances are included in the multidisciplinary approach. As such, early assessment by a surgeon and radiation oncologist is imperative in all patients with stage II/III NSCLC so the appropriate treatment algorithm can be selected with the patient and definitive treatment delivered in a timely manner. This again emphasizes the central role of the MDT helping form consensus on the patient’s therapeutic options.

However, the content of these discussions and potentially the therapeutic decisions are rapidly evolving. Firstly, data from the Checkmate 816 study revealed that a similar number of patients in the chemotherapy and immuno-chemotherapy proceeded to surgery. Of the 16% of patients that failed to proceed to surgery, just 1% of patients failed to undergo operative intervention because of complications with their neoadjuvant therapy [67]. These data highlight the safety of adding novel therapies in the neoadjuvant setting. The incidence of treatment-related adverse events also does not seem to change substantially with the addition of immunotherapy. Beyond the favorable safety profile, a recent meta-analysis of emerging data for patients with NSCLC treated with immuno-chemotherapy found nodal downstaging to occur in 71.9% of patients with N2 disease and ypN0 being found in 60.2% [68]. Adding immunotherapy to chemotherapy in stage II/III NSCLC has also contributed to a 6–9% increase in the R0 resection rate [67]. The pending results from the Neotorch study will shed further light specifically on those with stage III disease [69].

A similar improvement in the rate of R0 resections was also noted in the recently published TD-FOREKNOW [70]. Lei et al. randomized 94 patients with N2 disease (stage IIIA/IIIB) to receive either neoadjuvant camrelizumab plus platinum-based chemotherapy or chemotherapy alone. In total, an R0 resection was achieved in 92.5% of the treatment arm despite a high disease load. Furthermore, among patients treated with immuno-chemotherapy, pathological complete response occurred in 32.6% versus 8.9% in those who received chemotherapy alone. Whilst the secondary endpoints of event-free and disease-free survival were not met, the incidence of pCR was not dependent on PDL-1 status (PDL-1 < 1%: odds ratio 9.3, 95% CI 0.5–523.5; PDL-1 > 1%: odds ratio 5.8, 95% CI 0.8–67.4). Importantly, PDL-1 status was not available for over half of the patients and the rate of lymph node sterilization was not described. The NADIM II trial explored the outcomes in a similar group of patients finding that the addition of neoadjuvant nivolumab to chemotherapy resulted in more patients receiving definitive surgical therapy (91% vs. 69%) [71]. Moreover, a trend towards improved overall survival at 24 months was found in patients treated with neoadjuvant immuno-chemotherapy compared to chemotherapy alone (85.0%, 95% CI 75.9–95.2 vs. 63.6%, 95% CI 47.8–84.6) with a hazard ratio of death of 0.43 (95% CI 0.19–0.98).

Despite these impressive results among patients whom some would not consider eligible for surgical resection, it must be stated that the goal of neoadjuvant systemic therapy is not to render a non-resectable patient resectable. Conversely, it is important to recognize that neoadjuvant immunotherapy may change the landscape of who will undergo surgery. Interestingly, the finding that neoadjuvant durvalumab, when used in addition to SBRT, can achieve a major pathologic response in 53.5% versus 6.7% in durvalumab monotherapy may also alter the nature of the neoadjuvant therapy given when combined with radiation [72]. Although the attrition to surgery was 87% within this study, data from the real-world setting are lacking. This is particularly pertinent in the context of Pancoast tumors. Whilst induction chemoradiotherapy is the current standard of care based on non-randomized data [73], there is an absence of evidence beyond case reports that have explored the added role of immunotherapy in these challenging cases [74]. Further research is also urgently needed in this specific area, particularly because of the high rate of R1 resection, distant disease relapse and morbidity associated with resection.

It is important to note that for patients with stage II/III NSCLC, whilst there is a growing evidence base surrounding the use of novel drugs, no single regimen has yet been shown to outperform the other. When comparing between the trials of neoadjuvant immuno-chemotherapy, a similar number of patients proceed to surgery implying that the number of patients who suffer from functional decline during neoadjuvant treatment such that a patient is rendered inoperable is low. Data from the trial setting are limited by only including those with a good performance status at diagnosis. Real-world data are lacking and are required to understand the impact of different neoadjuvant systemic regimens in patient populations with lesser functional and physiological reserve.

Such real-world data are particularly relevant considering the rapidly expanding number of regimens available. Checkmate 816 utilized three cycles of platinum-based doublet immuno-chemotherapy and quickly became an accepted standard of care [67]. While other studies using two to four neoadjuvant cycles of immuno-chemotherapy with or without adjuvant systemic treatment have been described, the metrics of disease response and survival appear similar [71,75]. For instance, the recently reported randomized Keynote-671 study explored the role of a perioperative regimen of cisplatin-based chemotherapy with pembrolizumab or placebo [76]. With a pCR rate of 18.1% and 24-month disease free survival of 80.9%, these results are similar to other neoadjuvant immuno-chemotherapy strategies. Of note, whilst the addition of neoadjuvant ipilimumab improved tumor response over neoadjuvant nivolumab monotherapy, this seems to come at a cost of more than 30% of patients suffering grade 3–4 complications [77]. Considering this, and no clear oncological or survival benefit from additional agents or cycles, it is difficult to recommend additional treatment cycles with no gross benefit based on currently available data.

Patients with ALK/EGFR alterations were excluded from several studies investigating the use of neoadjuvant immuno-chemotherapy. As such, these regimens cannot be recommended for these specific patients. Although data from the Keynote-091 study suggested that adjuvant pembrolizumab may be associated with a trend towards improved survival in patients with resected EGFR mutant tumors [78], adjuvant immunotherapy beyond osimertinib has also not been found to provide a meaningful survival benefit in these specific patients [78,79]. Results from the ALINA study of adjuvant alecitinib plus platinum-based chemotherapy among patients with resected ALK-rearranged NSCLC are eagerly awaited [80].

The addition of PDL-1 inhibitors to traditional chemotherapy regimens in several trials has been associated with a higher rate of pathological complete response (pCR) as PD-L1 tumor expression increases (PD-L1 >50%, odds ratio of pCR = 44.7, 95% CI 28.6–61.7 vs. PD-L1 < 1% = 16.7, 95% CI = 9.2–26.8) [67]. Nevertheless, considering that data have also shown neoadjuvant PDL-1 inhibition to engender a survival benefit even if PDL-1 is <1%, based on current data, it is not clear how this should be used to inform neoadjuvant treatment decisions.

### 4.3. Perioperative Considerations

As reflected in the statements listed in Table 3, restaging of disease following neoadjuvant therapy is a crucial step in the treatment pathway with both contrast-infused CT or PET/CT providing adequate radiographic information [81]. However, early reports following neoadjuvant immuno-chemotherapy revealed some patients showed changes suggestive of radiological progression following treatment that were not subsequently supported by pathological findings. Termed pseudoprogression, this phenomenon is defined by an increase in size or avidity of the primary lesion or adjacent lymph nodes and represents an inflammatory rather than infiltrative process. Cascone and colleagues described these changes as ‘nodal flare’ characterized by nodal immune cell infiltration and granuloma formation [77].

Whilst this is a not an uncommon problem, the incidence varies among reports. Of the 20 patients enrolled into the Checkmate 157 trial, there were 2 patients had disease progression on their restaging CT scan despite having major pathological response to neoadjuvant nivolumab [82]. However, data from larger retrospective studies report a frequency of 2–4% [83,84].

The differentiation of pseudoprogression from true progression or hyperprogression is essential as pseudoprogression associated with improved disease survival and hyperprogression with more dismal outcomes [85]. However, a delay in treatment whilst additional investigations are performed could also negatively affect survival outcomes. Therefore, should progression be suspected during restaging, the results of PET/CT and CT imaging must be interpreted with caution and progressive disease urgently ruled out to determine the most appropriate therapy.

With regards to the modality chosen for restaging, limited access may prevent repeat PET/CT being performed; however, whether this leads to a difference in the incidence of missed disease progression is not clear. Comparative data from the setting of neoadjuvant immuno-chemotherapy are yet to be described. Following neoadjuvant chemoradiotherapy for patients with N2 disease, PET/CT missed residual N2 disease in 20% of patients compared to 25% following contrast-infused CT [86]. Thus, in settings where PET/CT access is limited, the use of a contrast-infused CT will likely suffice given the low likelihood of non-curative surgical interventions being performed using this imaging modality alone.

The improved diagnostic sensitivity of PET/CT over mediastinoscopy following neoadjuvant chemotherapy has been demonstrated [87]. However, these results are not generalizable to patients treated with immunotherapy and recent series lack granularity to meaningfully assess this further. Nevertheless, the incidence of missed disease on both CT and PET/CT seems to be low, and the use of both modalities is only likely to prevent a few cases of non-curative surgical interventions being performed.

For the 1–5% of patients who experience disease progression while induction chemotherapy or immuno-chemotherapy is administered [67,88,89], the ideal treatment regimen is not clear and several therapeutic options exist. Surgical resection is not appropriate if distant metastases become apparent or disease progression renders the lesion non-resectable. However, considering the limitations of restaging imaging noted above, surgical exploration may still be warranted in specific cases such as pseudo-progression with non-diagnostic endoscopic lymph node biopsy. Persistent N2 disease is not a contraindication to surgery provided it remains resectable.

If disease progression is suspected or confirmed, urgent re-consultation with the multidisciplinary team is mandatory and must include reassessment of the patient’s physiological status to ensure that surgery is feasible. The complexity of these decisions warrants an experienced thoracic surgeon to assess for operability and resectability in a center with sufficient operative volume to have had experience in managing these specific cases. This is particularly important considering the perceived increase in complexity of performing surgery after neoadjuvant immuno-chemotherapy [90]. If progressive or non-resectable disease is confirmed chemoradiation with consolidation immunotherapy may be the most suitable option based tentatively on data from the PACIFIC study [91].

In the Keynote 617 study, surgery was not performed in 17.9% of patients in the pembrolizumab arm. Although data regarding physical decline are not reported, 16/397 patients (4.0%) from the treatment arm did not undergo surgery due to a physician-led decision in the absence of clinical or radiological progression [76]. Failure to progress to surgery due to adverse outcomes related to neoadjuvant immuno-chemotherapy is uncommon, occurring in 1–6% of patients, similar to those receiving non-immunotherapy-based regimens [67,71,76]. If a patient is deemed physiologically unable to undergo surgery, as with progressive disease, chemoradiation with or without immunotherapy should be considered.

In patients who do undergo surgery, there are two important aspects of the proposed statements that warrant discussion. Firstly, although lymph node sampling of three mediastinal nodal stations and one hilar lymph node stations is mandatory in all patients undergoing surgery for stage II/III NSCLC, nodal dissection is preferable, particularly for clinically suspicious lymph node basins. However, it is important to recognize that whilst dissection is as safe as sampling, evidence varies with regards to whether dissection confers a survival advantage [92,93,94]. This evidence base is limited as only a few patients included in these studies had clinical stage II/III disease and none was treated with neoadjuvant immunotherapy.

Furthermore, whilst some advocate for lobe-specific nodal dissection based on the location of the primary lesion in stage I/II disease, such an approach has not been meaningfully tested in the setting of neoadjuvant systemic therapies [95]. For such an approach to apply, lymph nodes need to be clinically negative on initial staging investigations and non-suspicious intra-operatively. However, surprise upstaging to pN2 disease occurs in approximately 7%, questioning the validity of such an approach [96]. Moreover, considering that malignancy within the highest resected lymph node is considered an R1 resection and associated with a poorer prognosis, surgeons should be encouraged to pursue a thorough, rather than specific, mapping of mediastinal nodes.

Taken together, among recipients of neoadjuvant immuno-chemotherapy obtainment of an R0 resection may be optimized by the addition of a complete nodal dissection. This should achieve the most accurate pathological staging and reduction of residual nodal disease with a view to both improved prognostication and mitigation of locoregional disease recurrence. Therefore, in the absence of randomized data, we suggest that in stage II/III NSCLC following neoadjuvant therapy, systematic nodal dissection rather than sampling be performed. Further high-quality research in this area is needed, particularly in the setting of neoadjuvant immunotherapy, in order to underpin the recommendation made here.

Irrespective of the nodal dissection strategy, an anatomical lung resection should be performed. Lobectomy is considered the standard of care since the Lung Cancer Study Group trial [97]. A conservative approach to pneumonectomy may be required given the finding that in patients with N2 disease treated either with neoadjuvant or definitive chemoradiotherapy as part of the INT0139 trial, pneumonectomy was not associated with a survival benefit^61^. Furthermore, whilst the debate regarding pneumonectomy after concurrent chemoradiotherapy in this subset of patients is mature, its conduct after neoadjuvant immunotherapy is less well described [98]. For example, pneumonectomy was performed in just 17% of patients in Checkmate 816 with no subgroup analysis of complications. Preliminary 3-year event-free survival data from a subgroup analysis of the Checkmate study patients seem to suggest that excellent results can be achieved in well-selected patients treated with neoadjuvant immuno-chemotherapy and pneumonectomy (67% versus 48%) [99]. As such, we suggest that that pneumonectomy can be performed with a particular effort to minimize morbidity and mortality.

There are currently no data addressing the role of segmentectomy following neoadjuvant immuno-chemotherapy. Real-world data from a study of a national database suggest that segmentectomy performed for stage IA NCSLC was associated with an increased risk of R1 resection, substandard lymphadenectomy, increased local recurrence and, as a result, inferior overall survival in comparison to lobectomy [100]. Conversely, data from large trials underpin the oncological efficacy of segmentectomy in terms of survival [101,102]. Therefore, segmentectomy can theoretically be performed if there was no cN+ disease before the initiation of treatment, adequate gross and microscopic margins can be achieved and with intraoperative pathological assessment ruling out occult lymph node metastases. Such an approach is also supported by the finding that lobectomy does not confer a survival advantage compared to segmentectomy if unsuspected nodal upstaging occurs and if adjuvant therapy is given [103]. Although including patients with stage II/III disease, extrapolating these findings suggests that adjuvant therapy may be equally effective in more advanced stage disease too.

If on intraoperative frozen section performed during segmentectomy, the surgical margin is positive or metastatic nodal disease is found in the resected nodes, then completion lobectomy must be performed. Nevertheless, both segmentectomy and wedge resection accounted for just 2/325 patients recruited to the study arm of Keynote 617. As such, although technically possible in specific cases, there is insufficient evidence to support a resection less than lobectomy following neoadjuvant immuno-chemotherapy. In the absence of randomized data, we strongly suggest lobectomy is the treatment of choice for patients with stage II/III NSCLC. Segmentectomy should only be considered in the exceptional circumstance that poor lung function prevents a formal lobectomy.

These limitations highlight the need for intraoperative communication between the pathologist and surgeon to decide whether a frozen section is needed based on the proximity of the lesion to the surgical margins, clinical suspicion of metastatic lymphadenopathy and whether the operative approach would be changed with the pathological result. Although data regarding the accuracy of intraoperative frozen section following neoadjuvant immunotherapy are sparse, the incidence of false-negative frozen section appears low at around 1% in older series [104]. A precise intraoperative diagnosis has been shown to be an effective method to guide the extent of resection [105].

### 4.4. Postoperative Management

The consensus statements and level of recommendation regarding postoperative management are described in Table 4. The delivery of neoadjuvant therapy for patients with NSCLC has presented new challenges for the pathological assessment of surgical specimens. Considering the different mechanism of action of neoadjuvant immunotherapy versus chemotherapy, changes in the qualitative analysis of residual viable tumor were necessary [106]. This is due, in part, to accentuated inflammation, fibrosis and necrosis following the administration of neoadjuvant immunotherapy [107,108]. However, the histopathological changes associated with neoadjuvant therapy can also be found in treatment naïve tissues. Whilst this suggests that the ‘regression bed’ should be distinguished from the ‘tumor bed’, it is challenging to differentiate between the two and therefore in the interest of simplicity the International Association for the Study of Lung Cancer (IASLC) guidelines suggest using only the latter [109]. This refers specifically to the area that the pre-treatment lesion was located. Importantly, these guidelines also suggest a synoptic template by which pathology reports should adhere to and feature in several of the more recent clinical trials providing a common language between studies.

Importantly, there are areas of Canada and specific population groups in which the prevalence of tuberculosis (TB) is higher [110]. Whilst active TB infection is a contra-indication to neoadjuvant systemic therapies, super-infection with previously undiagnosed, dormant disease is theoretically possible [111].

In patients who did not receive neoadjuvant therapy, adjuvant chemotherapy has been associated with a survival benefit of 4.1–15% at 5 years [11,112,113,114]. Furthermore, randomized data have shown that adjuvant immuno-chemotherapy for resected NSCLC outperforms adjuvant chemotherapy in terms of survival outcomes. Approved regimens include cisplatin based chemotherapy with atezolizumab (Impower010) [79] and pembrolizumab (Keynote 091) [78] with other adjuvant chemotherapy trials are still recruiting. Much of this data is yet to mature but specifically in the Impower010 study, disease-free survival significantly improved if PDL-1 was >1% from 48% to 60% (HR 0.66, 95% CI 0.50–0.88, *p* = 0.039), seemingly greater than the improvement when PDL-1-negative patients were included too (49% to 56%, HR 0.79, 95% CI 0.64–0.96, *p* = 0.020). Moreover, subgroup analysis revealed that most of the survival benefit was derived from patients with PDL-1 >50%.

Evidence regarding the use of adjuvant pembrolizumab is somewhat controversial. Whilst the study group seemed to benefit from the addition of targeted therapy (disease free survival of 53.6 months, 95% CI 39.2-upper limit not met versus 42.0, 95% CI 31.3-uppper limit not met, hazard ratio 0.76, 95% CI 0.63–0.91, *p* = 0.0014), this survival benefit was not found in a subgroup analysis of patients with a PD-L1 CPS of >50% [78].

Therefore, whilst adjuvant atezolizumab is licensed for use in all patients undergoing resection of stage Ib–III NSCLC, in Europe and Canada, its use is restricted to those with a PDL-1 of >50%. Conversely, pembrolizumab is a valid alternative and is currently available in Canada through a compassionate-access program. Whilst currently there is no evidence suggesting that one regimen outperforms the other, the maturation of survival data may further define the role of adjuvant pembrolizumab.

Similarly, there is also currently no evidence comparing neoadjuvant versus adjuvant immuno-chemotherapy regimens in patients with resectable or resected stage II/III NSCLC. Whilst retrospective data suggest that neoadjuvant chemotherapy may be better tolerated, this does not translate into a disease-free or overall survival benefit [115]. This is reflected in data from the Impower010 study in which 172/495 patients randomized to receive adjuvant atezolizumab had treatment discontinued, a majority of whom were for adverse events [79].

Based on current evidence, there are specific settings in which adjuvant therapy is preferred, specifically in the setting of patients with an EGFR mutation where adjuvant tyrosine kinase inhibitors are associated with the biggest improvement in disease-free and overall survival. The third-generation osimertinib was shown in the ADAURA trial to improve 2-year DFS from 85 to 98% among patients with EGFR-mutated stage II/III NSCLC [116]. Moreover, recently presented data demonstrate that treatment with adjuvant osimertinib is associated with an increase in 5-year overall survival of 85% versus 73% among patients treated with placebo [117]. Although the authors suggest that adding adjuvant chemotherapy to osimertinib did not improve survival outcomes, a majority of patients who only received osimertinib had earlier stage disease (stage Ib, 75%), whilst a majority of patients with stage III disease received chemotherapy (stage IIIA, 81%). This could underestimate the perceived benefit of adding chemotherapy, and as such, for patients with stage II/III resected NSCLC with EGFR mutation, we strongly suggest adjuvant chemotherapy be given in addition to osimertinib. Phase II trial data have shown the safety of neoadjuvant osimertinib and a randomized phase III trial has recently started enrolling patients [118].

Irrespective the treatment modality chosen, there is currently no randomized evidence to guide adjuvant systemic treatment decisions based on response to neoadjuvant immuno-chemotherapy. This is particularly pertinent considering that 2-year event-free survival seems similar when comparing the neoadjuvant Checkmate 816 and the perioperative Keynote 671 regimens. Nevertheless, the finding that the event-free survival for recipients of perioperative pembrolizumab was improved when comparing those with and without major pathological response suggests that adjuvant therapy may be of benefit beyond neoadjuvant therapy alone. Whilst this specific point is an important area of future research, the discussion of the pathological results in the multidisciplinary meeting is nevertheless important for continuity of care, identifying changes in the patient’s physiological state should a perioperative regimen have been chosen, discussion regarding the risk of disease recurrence and the potential need for any further treatment to be given. Evidence regarding the use of adjuvant radiotherapy following neoadjuvant immuno-chemotherapy is sparse and as such those with positive margins require discussion in the MDT setting.

## 5. Conclusions

The treatment paradigms for patients with stage II/III NSCLC are rapidly changing. As our assessments for these patients become increasingly precise in terms of staging modalities and tumor biology, our MDTs are faced with more complex data to integrate as we attempt to orient our patients towards the most efficacious treatment strategy. The most efficacious regimen will include choices about combination of therapeutic modalities, sequencing and selection of individualized approaches within each subspecialty. The richness of these individual patient assessments means that the available trial data will not always provide the ideal guidance for regimen selection, certainly with a significant lack of comparative data across the systemic, radiation and surgical techniques that are available to us. This inherently makes treatment decisions more complex and it is hoped that the statements provided here will guide clinicians during a time when much of the data supporting these changes are still in flux and maturing. Further gaps in research have also been highlighted above with much of the new data leaving many questions that are fertile ground for future studies. We emphasize the need for treatment decisions to originate from the multidisciplinary team with a particular importance of placing the patient’s goals of care at the top of the decision tree. To achieve this, early referral to expert treating teams of physicians who can provide definitive treatment is mandatory so multi-stakeholder informed decisions can be made.

## Figures and Tables

**Table 1:** Consensus recommendations concerning diagnosis and staging.

Statement	Class of Recommendation	Level of Evidence
For patients with clinical stage II/III NSCLC considered for curative intent treatment, baseline staging investigations should include CT chest and upper abdomen with IV contrast, PET CT, minimally invasive mediastinal staging, and MRI or CT of the brain.	Class 1 (strong)	Level B-R
Co-ordination and communication between relevant care providers which may include a specialist nurse, helps ensure timely, appropriate, judicious and coordinated use of investigative resources to those that will affect the trajectory of patient care.	Class IIa (moderate)	Level C-LD
Treatment decisions for patients with NSCLC should be reviewed by a multidisciplinary group once staging investigations are completed.	Class I (strong)	Level B-NR
Biomarker testing should be performed at the time of diagnosis in order to direct neoadjuvant therapy. At a minimum, testing for EGFR, ALK and ROS1 and PD-L1 should be performed. More extensive testing for other actionable mutations using NGS is preferable.	Class I (strong)	Level B-R
The suitability for resection should be assessed prior to the initiation of neoadjuvant treatment and depends on a clinical assessment of the patient’s physiological reserve; medical comorbidities; anatomical feasibility of achieving an R0 resection based on pre-treatment imaging; and consent of the patient to undergo pulmonary resection after a balanced discussion regarding treatment alternatives.	Class I (strong)	Level B-NR

**Table 2:** Consensus recommendations regarding neoadjuvant treatment options.

Statement	Class of Recommendation	Level of Evidence
For patients deemed physiologically unsuitable, who decline surgical resection or for whom an R0 resection may not be possible due to borderline resectability or N2 disease, timely consultation with a radiation oncologist ensures consideration of chemoradiotherapy with consolidation immunotherapy as an alternative treatment with curative intent.	Class I	Level B-R
Patients with Pancoast tumors should be considered on a case-by-case basis based on the resectability of the lesion, the physiological reserve of the patient and the morbidity of various treatment modalities. Whilst the current standard of care includes chemoradiotherapy followed by surgical resection, neoadjuvant chemo-immunotherapy may be considered as an alternative option.	Class IIa	Level B-NR
At present, for patients with clinical stage II/IIIA NSCLC amenable to surgical resection who have sufficient physiological reserve, three cycles of neoadjuvant platinum-based chemotherapy doublet in combination with immunotherapy is the preferred neoadjuvant regimen if EGFR and ALK alterations have not been detected, irrespective of PD-L1 status. The relative benefit of neoadjuvant therapy options should be communicated to the patients at the time of consultation.	Class I	Level C-EO
Platinum doublet-based chemotherapy may be administered in the neoadjuvant or adjuvant setting for patients with a contraindication to immunotherapy.	Class IIa	Level B-R
In the presence of EGFR or ALK alterations, neoadjuvant chemo-immunotherapy is not recommended.	Class IIa	Level B-R

**Table 3:** Consensus recommendations regarding perioperative care.

Statement	Class of Recommendation	Level of Evidence
Following completion of neoadjuvant therapy, repeat CT thorax including upper abdomen and abdomen is mandatory, but PET CT is a valid alternative.	Class I (strong)	Level B-NR
If disease progression is detected following completion of neoadjuvant therapy, the case should be presented to the multidisciplinary team to evaluate the need to confirm the progression with consideration given to repeating invasive investigation and decide the subsequent therapy adapted to the clinical situation.	Class I (strong)	Level C-LD
If during neoadjuvant therapy physiological decline renders a patient unsuitable for surgical resection, definitive chemoradiotherapy and consolidation immunotherapy should be considered according to patient tolerance.	Class I (strong)	Level C-LD
Assessment by an experienced surgeon after restaging is required to assist with operative planning and identify the potential requirement for advanced pulmonary surgical management.	Class IIb (weak)	Level C-EO
Nodal dissection and sampling should at least include clinically or radiologically positive lymph nodes and a minimum of 1 hilar and 3 mediastinal lymph node stations.	Class I (strong)	Level B-R
Anatomical pulmonary resection (lobectomy, bilobectomy, pneumonectomy and extended lobectomy) are the preferred approaches to achieve an R0 resection following neoadjuvant chemo-immunotherapy. If the pulmonary function tests are borderline, an anatomical sublobar resection with wide margins can be considered in selected cases.	Class I (strong)	Level A
Intraoperative pathological assessment with or without frozen examination based on the pathologist’s evaluation and discussion with the surgeon should be performed to ensure an R0 resection has been achieved as this may modify the surgical approach.	Class I (strong)	Level B-NR
At present, patients with complete radiological response should still undergo resection but be carefully counselled regarding the possibility of having no tumor in the resected specimen.	Class I (strong)	Level C-EO

**Table 4:** Consensus recommendations regarding postoperative management.

	Level of Recommendation	Level of Evidence
Surgical pathology report for patients treated with neoadjuvant chemo-immunotherapy should include, over standard pathological assessments, a determination of pathological complete response according to the IASCLC-recommended protocol, ypTNM status, percent residual viable tumor and/or any appropriate/relevant biomarker testing not performed initially.	Class I (strong)	Level C-EO
All NSCLC patients with resected stage II/III who did not receive neoadjuvant therapy should be referred to medical oncology for discussion of adjuvant systemic chemotherapy, and adjuvant immunotherapy. All patients with resected EGFR mutant lung cancer should be also evaluated for chemotherapy and adjuvant third-generation tyrosine kinase inhibitor.	Class I (strong)	Level A
There are currently no randomized data supporting the use of adjuvant systemic chemotherapy or immunotherapy based on pathological response to neoadjuvant treatments and surgery.	Class IIa (moderate)	Level C-LD
Postoperative radiotherapy should be considered if a positive resection margin is found in the final pathological analysis on a case-by-case basis and discussed by the multidisciplinary team	Class IIa (moderate)	Level B-R

## Data Availability

The data presented in this study are available on request from the corresponding author.
